# Design and Characterization of a Cheese Spread Incorporating *Osmundea pinnatifida* Extract

**DOI:** 10.3390/foods12030611

**Published:** 2023-02-01

**Authors:** Margarida Faustino, Daniela Machado, Dina Rodrigues, José Carlos Andrade, Ana Cristina Freitas, Ana Maria Gomes

**Affiliations:** 1 Universidade Católica Portuguesa, CBQF—Centro de Biotecnologia e Química Fina—Laboratório Associado, Escola Superior de Biotecnologia, Rua Diogo Botelho 1327, 4169-005 Porto, Portugal; 2 TOXRUN-Toxicology Research Unit, University Institute of Health Sciences, CESPU, CRL, 4585-116 Gandra, Portugal

**Keywords:** antidiabetic, antihypertensive, antioxidant, food, microbiological quality, *Osmundea pinnatifida*, prebiotic, whey cheese, yogurt

## Abstract

Marine algae have been emerging as natural sources of bioactive compounds, such as soluble dietary fibers and peptides, presenting special interest as ingredients for functional foods. This study developed a cheese spread incorporating red seaweed *Osmundea pinnatifida* extract and subsequently characterized it in terms of nutritional, pH, and microbiological parameters and bioactivities including prebiotic, antidiabetic, antihypertensive, and antioxidant activities. This food was produced through incorporation of *O. pinnatifida* extract (3%), obtained via enzymatic extraction Viscozyme L in a matrix containing whey cheese (75%) and Greek-type yoghurt (22%). The product was then subjected to thermal processing and subsequently stored for 21 days at 4 °C. During storage, this food showed a high pH stability (variations lower than 0.2 units), the absence of microbial contamination and all tested bioactivities at the sampling timepoints 0 and 21 days. Indeed, it exerted prebiotic effects under *Lactobacillus acidophilus* LA-5^®^ and *Bifidobacterium animalis* subsp. *lactis* BB-12^®^, increasing their viability to around 4 and 0.5 log CFU/g, respectively. In addition, it displayed antidiabetic (α-glucosidase inhibition: 5–9%), antihypertensive (ACE inhibition: 50–57%), and antioxidant (ABTS: 13–15%; DPPH: 3–5%; hydroxyl radical: 60–76%) activities. In summary, the cheese spread produced may be considered an innovative food with high potential to contribute toward healthier status and well-being of populations.

## 1. Introduction

Increasingly, the public’s attention has focused on nutrition, health, and well-being, and the dietary behaviors have changed; with this aim, more consumers are seeking more natural and healthier food products [1]. Consequently, novel food sources, such as seaweeds, have emerged as valuable candidates for design of new nutrient-rich added-value foods. In fact, seaweeds present an interesting nutritional profile with soluble dietary fibers, peptides, phlorotannins, lipids, and minerals as major compounds and simultaneously comprise a diversity of health-promoting compounds [2,3,4]. Among the seaweeds, *Osmundea pinnatifida* (belonging to Rhodophyta phylum) is commonly described as an edible red macroalga to be incorporated into food or as a supplement, frequently encountered wild in European coasts or eventually cultivated in IMTA systems (Algaplus, Portugal) or more recently in the Algem^®^ photobioreactor system and with relevant bioactivities in terms of antioxidant, antidiabetic, and prebiotic properties [3,4,5,6].

Parallelly, dairy-fermented products, namely milk, cheese, and yogurt, have been extensively explored as carriers for bioactive compounds; given their combined effect—high nutritional value, they are a source of energy and key nutrients, namely vitamins and minerals, and exert health benefits, since they contribute to gastrointestinal well-being maintenance and improvement of the immune system [7]. Among the different yoghurt types, the Greek-type yogurt, which is obtained after draining the whey, leading to higher total solids and lower lactose contents than regular yogurt, offers an interesting potential to be used as an ingredient in product development given its attractive sensory properties [8]. From a circular economy perspective, whey cheeses have gained prominence in the last decades as a sustainable food matrix for bioactive ingredients incorporation including probiotics and antihypertensive peptides [9,10,11,12]. Whey cheese is a traditional product manufactured via thermal processing of whey, leading to denaturation of its soluble proteins and locally known as “Requeijão” (Portugal), “Requéson” (Spain), or “Ricotta” (Italy) [12,13]. This type of soft cheese (“Requeijão”) contains several whey proteins, including α-lactalbumin, β-lactoglobulin, lactoferrin, lactoperoxidase, serum albumin, and glycomacropeptide, which presents important nutritional and health-related characteristics [11]. In addition, it is important to note that in recent studies involving spreadable cheeses it was demonstrated that their fortification with specific compounds, such as fruit and vegetable by-products, may increase certain bioactivities, namely antioxidant activity, or affect favorably metabolic biomarkers such as glucose and triglyceride levels [14,15].

By considering the beneficial nutritional profiles of yogurt and whey cheese and their potential as carriers for bioactive compounds, a novel approach was designed and applied. The traditional whey cheese (“Requeijão”) and the Greek-type yogurt were combined to produce a healthy, versatile food matrix for *O. pinnatifida* extract incorporation, obtaining a cheese spread. This innovative food was then characterized in terms of nutritional, pH, and microbiological parameters and bioactivities including prebiotic, antidiabetic, antihypertensive, and antioxidant properties.

## 2. Materials and Methods

### 2.1. Seaweed Extract Preparation

*Osmundea pinnatifida* specimens were collected in Buarcos bay (Figueira da Foz, Portugal), cleaned and dried according to Rodrigues et al. [4]. Afterwards, an enzymatic extraction of *O. pinnatifida* by Viscozyme L (Sigma-Aldrich, St. Louis, MO, USA) was carried out according to the protocol described by Rodrigues et al. [5]. Briefly, 2 g of dried seaweed in 50 mL of deionized water was incubated in a water bath with shaking at 50 °C for 10 min. After adjusting pH to specific enzyme optimum conditions (Viscozyme^®^ L, pH = 4.5 at 50 °C), 100 mg of enzyme was added and incubated at 50 °C during 24 h for enzymatic hydrolysis. The enzymatic reaction was then stopped by heating the sample at 90–100 °C for 10 min followed by immediate cooling in an ice bath. The enzymatic aqueous solutions were then centrifuged at 4000 rpm for 10 min (Universal 320R, Hettich, Tuttlingen, Germany), filtered in vacuum with a filter (diameter: 0.22 µm), frozen at −80 °C and lyophilized. The pH of all extracts was adjusted to pH 7.0 with 1M HCl and/or NaOH before freezing.

### 2.2. Cheese Spread Production

**Figure 1:**
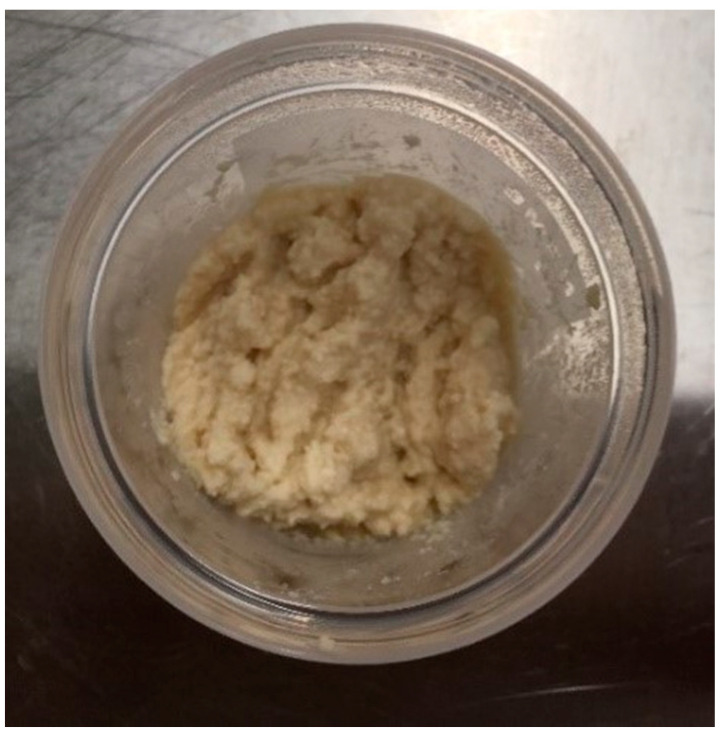
Photograph of the cheese spread produced incorporating *Osmundea pinnatifida* extract.

Commercial whey cheese from cow’s milk (“Requeijão”; protein: 9.5 g; lipids: 8.5 g; total sugars: 6.3 g) and a Greek-type yoghurt (without added sugar; protein: 3.9 g; lipids: 8.0 g; total sugars: 4.6 g) were purchased from the local supermarket (Continente, Porto, Portugal). The recipe for the cheese spread was based on 75% (*w*/*w*) of pasteurized whey cheese, 22% (*w*/*w*) of the Greek-type yoghurt, and 3% (*w*/*w*) of *O. pinnatifida* extract (abbreviated to CSExt). These ingredients were mixed and subjected to thermal treatment (90 °C for 30 min in a shaking water bath). Then, the cheese spread was divided into portions of 10 g and stored in sterile containers for 21 days under refrigerated conditions (4 °C; Figure 1). In addition, control cheese spreads were used in the present work, i.e., a cheese spread supplemented with 3% (*w*/*w*) fructooligosaccharides (FOS, Orafti^®^ P95; Orafti, Belgium) seaweed extract (abbreviated to CSFos) and a plain cheese spread without FOS or seaweed extract (abbreviated to CS) were prepared and stored under the same conditions. Three batches of each cheese spread (CSExt, CSFos, and CS) were prepared and analyzed.

### 2.3. Nutritional, pH, and Microbiological Parameters Evaluation

The nutritional values of the cheese spread were calculated based on the nutrition values for each of the constituent ingredients, namely whey cheese, Greek-type yoghurt, and *O. pinnatifida* extract and their respective specific weights in the formulation. This information was provided per 100 g of cheese spread. Furthermore, the pH and microbiological analyses of the cheese spread (CSExt, CSFos, and CS) were performed at the day of production (day 0) and after 7, 14, and 21 days of refrigerated storage. In the microbiological quality analysis, for each cheese spread at each sampling time, a 2.4 g aliquot of each sample was homogenized in 24 mL of sterile 0.1% (*w*/*v*) peptone water (Sigma-Aldrich, St. Louis, MO, USA) for 3 min in a Stomacher blender (Model 400 circulator, from Seward Laboratory Systems Inc, Worthing, UK). Sequential decimal dilutions of cheese spread homogenates were made with sterile 0.1% (*w*/*v*) peptone water and plated in duplicate, using Miles and Misra method [16], on the following solid media: de Man Rogosa and Sharpe agar (MRS, Biokar Diagnostics, Beauvais, France) for enumeration of lactic acid bacteria; a plate count agar (PCA, Merck, Darmstadt, Germany) for aerobic mesophilic bacteria detection and a violet red bile glucose agar (VRBGA, Biokar Diagnostics, Allone, France) for coliform bacteria and the enterobacteriaceae count. Incubation conditions of the inoculated agar plates were as follows: MRS, at 37 °C aerobically for 2 days; PCA, at 30 °C aerobically for 2–5 days; VRBGA, at 37 °C aerobically for 2 days. After the incubation of agar plates, the colony numbers were counted, and the results were expressed as the number of colony-forming units (CFUs) per gram (CFU/g) of cheese spread.

### 2.4. Prebiotic Activity Determination

The potential prebiotic activities of the cheese spread incorporating *O. pinnatifida* extract were determined at 0 and 21 days of storage by enumeration of viable cells of two probiotic strains following the procedure proposed by Rodrigues et al. [5] with some modifications. Briefly, commercial probiotic strains, i.e., *Lactobacillus acidophilus* LA-5^®^ (Chr. Hansen, Hoersholm, Denmark) and *Bifidobacterium animalis* subsp. *lactis* BB-12^®^ (Chr. Hansen, Hoersholm, Denmark), were inoculated at 2%, in an independent form and with duplicates, in the 3 different cheese spreads: (i) with 3% (*w*/*w*) of *O. pinnatifida* extract (CSExt); (ii) with 3% (*w*/*w*) FOS (positive control, CSFos); and (iii) plain cheese spread (negative control, CS). Then, the different inoculated cheese spreads were incubated at 37 °C aerobically for 24 h. During this period, the growth of probiotic strains was monitored, by plating CFUs in duplicate in MRS agar plates (for LA-5^®^ enumeration) and MRS supplemented with 0.05% (*w*/*v*) of cysteine-HCl (Sigma-Aldrich, St. Louis, MO, USA) agar plates (for BB-12^®^ enumeration) at timepoints of 0, 6, 12, and 24 h. The incubation conditions for agar plates were as follows: 37 °C for 48 h under aerobic conditions for MRS and at 37 °C for 48 h under anaerobic conditions generated in jars containing AnaeroGen ^TM^ 2.5 L sachet (Thermo Scientific, Waltham, MA, USA) for MRS with cysteine-HCl.

### 2.5. Sample Preparation for Antidiabetic, Antihypertensive, and Antioxidant Activities

Lyophilized cheese spread samples were prepared according to the procedure described by Apostolidis, Kwon, and Shetty [17] with minor changes. For antidiabetic and anti-hypertensive activities evaluation, 30 mg of each freeze-dried cheese spread was dissolved in 1 mL of 13 mM sulfuric acid and 1 mL of deionized water, respectively. For antioxidant activity assessment, 10 mg of each freeze-dried cheese spread was dissolved in 1 mL of deionized water. Homogenized freeze-dried samples were centrifuged at 14,000 rpm for 10 min at 4 °C.

### 2.6. Antidiabetic Activity Determination

The antidiabetic potentials of the cheese spreads (CSExt, CSFos, and CS) were measured through α-glucosidase inhibitory activity determination using 96-well microplates assay proposed by Kwon, Apostolidis, and Shetty with minor alterations [18]. Briefly, 50 μL of each homogenized cheese spread was mixed with 100 μL of 0.1 M phosphate buffer (pH 6.9) containing an α-glucosidase solution (1.0 U/mL) (Sigma-Aldrich, St. Louis, MO, USA) and pre-incubated at 25 °C for 10 min. Afterwards, 50 μL of 5 mM p-nitrophenyl-α-D-glucopyranoside (Sigma-Aldrich, St. Louis, MO, USA) solution in 0.1 M phosphate buffer (pH 6.9) was added to each well at 5 s intervals. The reaction mixtures were incubated at 25 °C for 5 min, and the absorbance readings were recorded at 405 nm by a multiscan microplate fluorometer (FLUOstar Optima; BMG Labtech, Offenburg, Germany) and compared to a control which had 50 μL of buffer solution, instead of a homogenized cheese spread. Acarbose (Sigma-Aldrich, St. Louis, MO, USA) was used as the positive control at a concentration of 10 mg/mL. The α-glucosidase inhibitory activity was expressed as the α-glucosidase inhibition percentage (%) and was calculated as follows:

(1) $$\alpha -\mathrm{Glucosidase}\;\mathrm{nhibition}\;\%=\left(\frac{∆A\;\mathrm{control}-∆A\;\mathrm{sample}}{∆A\;\mathrm{control}}\right)\times 100$$

where ΔA control is the variation of absorbance of the control and ΔA sample is the variation of absorbance of the samples. All assays were performed in triplicate.

### 2.7. Antihypertensive Activity Determination

The potentials of the antihypertensive cheese spreads (CSExt, CSFos, and CS) were carried out by measuring the angiotensin-I-converting enzyme (ACE) inhibition, using the fluorimetric method described by Sentandreu and Toldrá [19] and modified by Quirós et al. [20] with some alterations. Briefly, 160 μL of fluorescent substrate o-Abz-Gly-p-Phe(NO_2_)-Pro-OH at 0.45 mM (Bachem AG, Bubendorf, Switzerland) prepared in Tris buffer with 1.125 M of NaCl and 40 μL of homogenized cheese spread (CSExt, CSFos, and CS) was added in each microtiter-plate well (Nunc, Roskilde, Denmark). The enzyme reaction was initiated by the addition of 2 mU of ACE (Sigma-Aldrich, St. Louis, MO, USA), previously prepared in glycerol (50%) and stored at −20 °C in 200 µL aliquot. On the day of the measurement, the enzyme was prepared in a buffer solution of Tris-HCl (150 mM) with 0.1 mM de ZnCl_2_ (pH 8.3) that were immediately mixed and incubated at 37 °C. The generated fluorescence was measured after 30 min using a FLUOstar OPTIMA plate reader. The wavelengths used were 350 nm (excitation) and 420 nm (emission). The ACE inhibition was expressed as the ACE inhibition percentage (%) and was calculated as follows:

(2) $$\mathrm{ACE}\;\mathrm{Inhibition}\;\%=\left(\frac{\left(\mathrm{control}-\;\mathrm{blank}\right)-\left(\mathrm{sample}-\mathrm{sample}\;\mathrm{blank}\right)}{\left(\mathrm{control}-\mathrm{blank}\right)}\right)\times 100$$

where control wells contained 40 µL of MilliQ water instead of the sample, 40 µL of the ACE, and 160 µL of the substrate; blank wells contained 80 µL MilliQ water instead of the sample and without the ACE; sample wells contained 40 µL of the sample, 40 µL of the ACE, and 160 µL of the substrate, and sample blank wells were used to subtract the sample background and did not contain the ACE. All assays were performed in triplicate.

### 2.8. Antioxidant Activity Determination

The potential antioxidant activities of the cheese spreads (CSExt, CSFos, and CS) were measured via the following methods: (i) ABTS radical scavenging assay; (ii) DPPH free radical scavenging assay; and (iii) hydroxyl radical scavenging assay.

#### 2.8.1. ABTS Radical Scavenging Assay

The ABTS radical scavenging activity was determined according to the method described by Gião et al. [21]. Briefly, 120 μL of each homogenized cheese spread was added to 2 mL of a diluted ABTS solution and left to react for 6 min. After this, the absorbance at 734 nm (A sample) was measured in a UV-VIS spectrophotometer (Shimadzu, Barueri, SP, Brasil). Three replicates were performed. Using ascorbic acid as the standard (0 to 1 mg/mL), the results were expressed as the equivalent concentration of ascorbic acid (mg ascorbic acid equiv/mL). The percentage of the ABTS scavenging activity was calculated using the following formula:

(3) $$\mathrm{ABTS}\;\mathrm{Scavenging}\;\%=\left(\frac{A\;\mathrm{control}-A\;\mathrm{sample}}{A\;\mathrm{control}}\right)\times 100$$

For each sample, the initial absorbance of 2 mL of diluted ABTS solution was measured (indicated as A control).

#### 2.8.2. DPPH Free Radical Scavenging Assay

The DPPH free radical scavenging was quantified following the method proposed by Suresh et al. [22] with some modifications. Briefly, an aliquot (0.1 mL) of each homogenized cheese spread (CSExt, CSFos, and CS) was added to 3.0 mL of 0.1 mM ethanolic DPPH solution (Sigma-Aldrich, St. Louis, MO, USA), and absorbance were measured at 515 nm after incubation for 30 min at 30 °C in the dark (A sample) in a UV-VIS Spectrophotometer (Shimadzu). Three replicates were performed. Using trolox (Sigma-Aldrich, St. Louis, MO, USA) as the standard (0–0.05 mg/mL), the results were expressed as the equivalent concentration of trolox (mg trolox equiv/mL), being the DPPH free radical scavenging percentage calculated using the following formula:

(4) $$\mathrm{DPPH}\;\mathrm{Scavenging}\;\%=\left(1-\frac{A\;\mathrm{sample}-A\;\mathrm{blank}}{A\;\mathrm{control}}\right)\times 100$$

For each sample, the absorbance of 3 mL of DPPH plus 0.1 mL ethanol was measured as the control (A control), whereas the absorbance of 3 mL of ethanol plus 0.1 mL of homogenized cheese spread was measured as the blank (indicated as A blank).

#### 2.8.3. Hydroxyl Radical Scavenging Assay

The hydroxyl radical scavenging assay was performed according to the protocol proposed by Sudha et al. [23] and based on the previous work of Smirnoff and Cumbes [24]. Briefly, an aliquot (1 mL) of each homogenized cheese spread (CSExt, CSFos, and CS) was added to 2 mL of a reaction mixture containing 1 mL of 1.5 mM FeSO_4_ (Merck, Darmstadt, Germany), 0.7 mL of 6 mM hydrogen peroxide (Merck, Darmstadt, Germany), and 0.3 mL of 20 mM of sodium salicylate (VWR, Leuven, Belgium). After incubation for 1 h at 37 °C, the absorbance of the hydroxylated salicylate complex was measured at 562 nm (indicated as A sample) in a UV-VIS spectrophotometer (Shimadzu, Barueri, SP, Brasil). Three replicates were performed. Using ascorbic acid as the standard (0–1 mg/mL), results were expressed as the equivalent concentration of ascorbic acid (mg ascorbic acid equiv/mL), being the hydroxyl radical scavenging percentage calculated using the following formula:

(5) $$\mathrm{Hydroxyl}-\mathrm{radical}\;\mathrm{Scavenging}\;\%=\left(1-\frac{A\;\mathrm{sample}-A\;\mathrm{blank}}{A\;\mathrm{control}}\right)\times 100$$

For each sample, the absorbance of 2 mL reaction mixture plus 1 mL of deionized water was measured as the control (indicated as A control), whereas 2 mL of the reaction mixture with sodium salicylate replaced by water plus 1 mL of cheese spread was measured as the blank (indicated as A blank).

### 2.9. Stastical Analysis

Data were expressed as the mean ± standard deviation (SD) of replicates. Results from prebiotic, antihypertensive, and antioxidant activities were analyzed by one-way ANOVA in association with Tukey’s multiple comparison test (post-hoc test), since all data followed a normal distribution according to the Shapiro−Wilk’s Test. All tests were performed with a significance level of 5% (*p* < 0.05) and using the Statistical Package for Social Sciences software (version 21; SPSS, Chicago, IL, USA).

## 3. Results and Discussion

### 3.1. Nutritional, pH, and Microbiological Parameters of Cheese Spreads

The proximate composition of the cheese spread containing *O. pinnatifida* extract reflected the basic composition of each ingredient used. The total protein and total carbohydrate contents were found to be 5.05 g/100 g each, and the total fat content was found to be 7.87 g/100 g. *O. pinnatifida* extract mainly contributed to the carbohydrate fraction (two-thirds of which are sulfated sugars [3]), although the protein content was also further enhanced by the extract addition. A good distribution between macronutrients was observed with an energy content of 111 kcal/100 g. The incorporation of *O. pinnatifida* did not affect greatly the final pH value of the cheese spread, lowering 0.3 units from 5.95 to 5.61 for the CS and the CSExt, respectively. The pH values were maintained throughout storage with no more than a 0.2-unit variation in the case of the CSExt and CS foods; in the case of the CSFos, the initial pH was slightly lower (5.46), and the variation throughout storage reached 0.6 units.

In terms of the microbiological quality, despite the long history of dairy products consumption by humans, their contamination by spoilage microorganisms and pathogens from the environment remains as one of the major matters of concern among food industries and populations. In fact, the microbial contamination of milk and derived products has been related with several foodborne outbreaks in many countries [25]. To overcome this, different thermal treatments have been proposed as a valuable strategy to prevent the microbial contamination, ensuring the microbiological quality and safety of end products [26]. Thus, the effectiveness of thermal processing (90 °C for 30 min in a shaking water bath) of the cheese spreads (CSExt, CSFos, and CS) to prevent microbial contamination during 21 days of storage was assessed. Our microbiological analysis showed that bacterial counts in all cheese spreads (CSExt, CSFos, and CS) were below the limit of detection of the CFU plating technique, i.e., lower than 500 CFU/g, in all sampling timepoints (0, 7, 14, and 21 days after refrigerated storage). Consequently, these results indicated that the novel developed cheese spread had a shelf-life of at least 21 days under refrigerated storage conditions.

Additionally, the cheese spread incorporating *O. pinnatifida* extract (CSExt) was preliminarily analyzed in terms of the sensory potential. No formal methodology was applied, due to limitations in product quantity, but preliminary testing enabled perceiving the promising acceptability of the product, since it was ranked very positively in terms of consumption intention and specific attributes, namely aspect, odor, flavor, creaminess, and consistency. Nevertheless, it is important that further trials are run using a large number of potential consumers and a properly designed methodology to fully characterize the sensory attributes of this new food product.

### 3.2. Prebiotic Activity of Cheese Spreads

**Figure 2:**
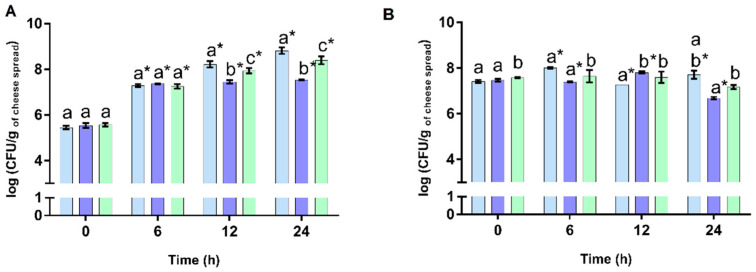
Evolution of viable cell numbers of *Lactobacillus acidophilus* LA-5^®^ (**A**) and those of *Bifidobacterium animalis* subsp *lactis* BB-12^®^ (**B**) in the cheese spread incorporated with *Osmundea pinnatifida* (CSExt, shown by the blue bar), the plain cheese spread (CS, shown by the purple bar), and the cheese spread with FOS (CSFos, shown by the green bar) upon manufacture (0 days), incubated over 24 h at 37 °C. Different letters indicate statistically significant differences (*p* < 0.05) between the three cheese spreads at each sampling time, while bars marked with * means statistically significant differences (*p* < 0.05) in comparison to the data obtained at 0 h.

In the recent years, the potential use of marine seaweeds or their extracts as prebiotics has gained a special focus among the scientific community and the food industry [27]. A prebiotic is defined as “a substrate that is selectively utilized by host microorganisms conferring a health benefit” and may be of different chemical nature as proposed by the International Scientific Association for Probiotics and Prebiotics (ISAPP) including dietary oligosaccharides, polyphenols, and eventually polyunsaturated fatty acids converted to respective conjugated fatty acids. Health benefits associated with prebiotics include gut bacteria modulation, reduction in blood lipid levels, effects upon insulin resistance, and mineral bioavailability enhancement [28]. In the present work, the potential prebiotic effects of the three cheese spreads (CSExt, CSFos, and CS), upon 0 and 21 days of refrigerated storage, were evaluated by using the pure probiotic cultures of *L. acidophilus* LA-5^®^ and *B. animalis* subsp. *lactis* BB-12^®^ that are representative beneficial bacterial strains of genera *Lactobacillus* and *Bifidobacterium*, respectively, usual target genera for prebiotics [5]. As can be seen in Figure 2A,B, the CSExt upon manufacture (day 0 of storage) exhibited a prebiotic potential, since a higher average values of log CFU/g were found in the CSExt than for the CSFos (positive control) and the CS (negative control) after 24 h of incubation for both probiotic bacteria. Indeed, *L. acidophilus* LA-5^®^ incubated in the CSExt increased viability around 2.8 log cycles between timepoints 0 and 12 h, reaching values of 8.22 log CFU/g of the cheese spread and increasing to 8.82 log CFU/g by 24 h of incubation (Figure 2A). Comparatively, *L. acidophilus* LA-5^®^ incubated in the CSFos (positive control) did not reach such high numbers reporting viable cell numbers of 0.28 and 0.43 log cycles lower at the timepoints 12 h and 24 h, respectively (Figure 2A). It should be noted that *B. animalis* subsp. *lactis* BB-12^®^ growth promotion by the CSExt was not as effective as for *L. acidophilus* LA-5^®^ (Figure 2B). Indeed, the *B. animalis* subsp. *lactis* BB-12^®^ viable cell numbers in the CSExt only increased by around 0.3 log cycles over the full 24-hour time framework, denoting a lower prebiotic effect. This phenomenon may be related to the large quantity of sulfated sugars observed in this extract. Red seaweeds, such as *O. pinnatifida,* have been usually depicted as an agar producer, and it is recognized that microorganisms are not able to hydrolyze and metabolize this polysaccharide, which is used as a solidifying agent of culture media [5]. On the other hand, it should not be completely ignored that some low-molecular-weight polysaccharides derived from agar and alginate seaweeds were fermented by gut microbiota, namely bifidobacterial populations, showing the potential to be used as a novel source of prebiotics [29]. Even so, the CSExt exerted a better prebiotic effect in *B. animalis* subsp. *lactis* BB-12^®^ than the other cheese spreads (the CS and the CSFos). Notably, our results are in agreement with the previous work of Rodrigues et al. that were the pioneers in demonstration of the prebiotic potential of the seaweed *O. pinnatifida* extract and who also reported a higher prebiotic effect in *L. acidophilus* LA-5^®^ than *B. animalis* subsp. *lactis* BB-12^®^ [5].

**Figure 3:**
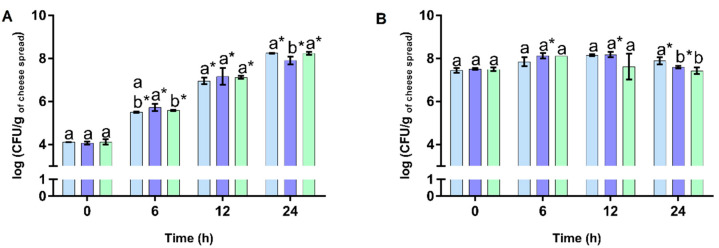
Evolution of viable cell numbers of *Lactobacillus acidophilus* LA-5^®^ (**A**) and those of *Bifidobacterium animalis* subsp *lactis* BB-12^®^ (**B**) in the cheese spread incorporated with *Osmundea pinnatifida* (CSExt, shown by the blue bar), the plain cheese spread (CS, shown by the purple bar), and the cheese spread with FOS (CSFos, shown by the green bar) after 21 days of refrigerated storage, incubated over 24 h at 37 °C. Different letters indicate statistically significant differences (*p* < 0.05) between the three cheese spreads at each sampling time, while bars marked with * means statistically significant differences (*p* < 0.05) in comparison to the data obtained at 0 h.

As previously referred, at 21 days upon storage, the prebiotic activities of the three cheese spreads (CSExt, CSFos, and CS) were tested to ascertain whether this specific bioactivity was maintained throughout storage. As depicted in Figure 3A,B, the CSExt stored for 21 days displayed prebiotic activity, since significant increments (*p* < 0.05) in viable cell numbers of *L. acidophilus* LA-5^®^ and *B. animalis* subsp. *lactis* BB-12^®^ incubated in the CSExt by 24 h were detected. Furthermore, the prebiotic activity of the CSExt was more pronounced for *L. acidophilus* LA-5^®^ than for *B. animalis* subsp. *lactis* BB-12^®^ following a similar trend to that reported in day 0 of storage (Figure 2).

### 3.3. Antidiabetic Activity of Cheese Spreads

**Figure 4:**
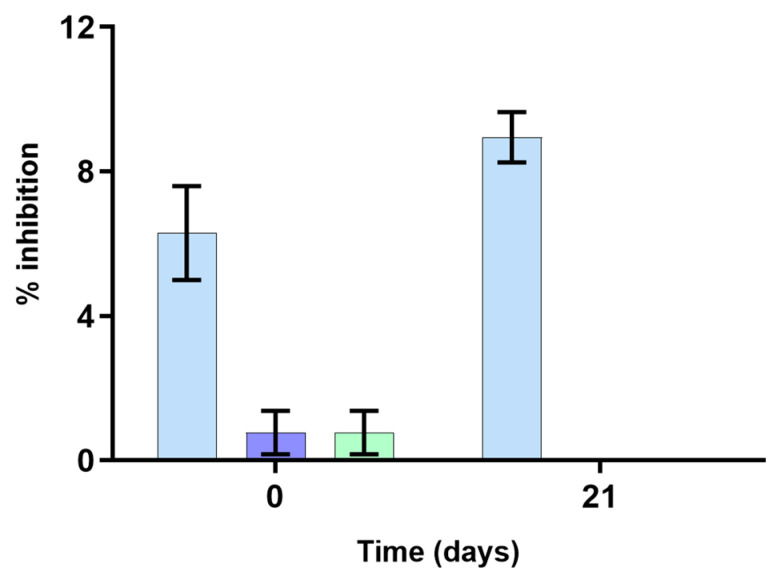
α-Glucosidase inhibitory activity, expressed as the inhibition percentage, of the cheese spread incorporated with *Osmundea pinnatifida* extract (CSExt, shown by the blue bar), the plain cheese spread (CS, shown by the purple bar), and the cheese spread with FOS (CSFos, shown by the green bar) at 0 and 21 days of refrigerated storage.

Diabetes mellitus is a global chronic disease with significant comorbidities and characterized by hyperglycemia and insulin resistance. An efficient therapeutic approach to controlling the glycemic level is to delay the absorption of glucose by inhibiting the activity of carbohydrate digestive enzymes such as α-glucosidase [30]. Remarkably, it has been proposed that seaweeds can be a promising source of α-glucosidase inhibitory compounds [31,32,33]. In this alignment, the α-glucosidase inhibitory activities of the cheese spreads (CSExt, CSFos, and CS) were evaluated. As can be observed in Figure 4, the CSExt displayed a higher α-glucosidase inhibitory activity than the CS and the CSFos in both sampling timepoints. Specifically, the CSExt exhibited 8.95% ± 0.70% of α-glucosidase inhibition at 21 days of refrigerated storage, whereas the CS and the CSFos displayed undetectable inhibition levels in the same sampling timepoints. Interestingly, Rodrigues et al. reported higher α-glucosidase inhibition levels for seaweed *O. pinnatifida* extract, namely 38–40% inhibition [5]. However, such inhibition levels were not achieved in the present work, probably due to the thermal treatment that *O. pinnatifida* extract underwent for CSExt production, which did not occur in the study of Rodrigues et al. [5]. Indeed, it is well established that food processing methods such as thermal processing may affect the biological activity of bioactive compounds present in added-value foods [34]. Furthermore, the delivery matrix may also interfere with the observed activity by means of entrapment of bioactive compounds hampering their enzyme inhibitory effects.

### 3.4. Antihypertensive Activity of Cheese Spreads

In the last years, the inhibition of angiotensin-I-converting enzyme (ACE) has become a major target for the control of hypertension, recognized as a worldwide health problem with epidemic proportions and one of the main risk factors for cardiovascular disease [35,36]. However, synthetic ACE-inhibitors cause several undesirable side effects [36,37]. As a natural alternative solution, several authors demonstrated that marine algae extracts and their components may exert antihypertensive effects [35,38,39,40,41]. Within this context, the ACE inhibition percentages were determined in the cheese spread incorporating *O. pinnatifida* extract (CSExt) and in cheese spreads used as controls (namely CS and CSFos) at 0 and 21 days of storage, and in *O. pinnatifida* extract for comparative purposes. As can be seen in Table 1, *O. pinnatifida* extract exhibited the greatest antihypertensive effect (with 68% of ACE inhibition), followed by the CSExt (around 50–57% of ACE inhibition). In contrast, the CS and the CSFos displayed undetectable ACE inhibition levels according to the limit of detection of ACE inhibition assays which corroborates the absence of contribution from the matrix itself. Furthermore, the antihypertensive activity of the CSExt was retained throughout the storage period, presenting around 57% of ACE inhibition at day 0 and a slight decrease to 50% of ACE inhibition after 21 days of storage (Table 1). To the best of our knowledge, the present study is the first evaluating antihypertensive activity of *O. pinnatifida* extract and their derived foods (i.e., CSExt). Nevertheless, it is important to note that antihypertensive activity has been reported for extracts from other red seaweeds. Indeed, Cha et al. screened the ACE inhibitory activities of methanolic and aqueous extracts prepared from 26 species of red seaweeds and found that *Lomentaria catenata* aqueous extract prepared at 20 °C exhibited the strongest ACE inhibitory activity with an inhibitory percentage around 99% [35].

### 3.5. Antioxidant Activity of Cheese Spreads

Seaweeds have been receiving increasing attention as a promising natural source of antioxidant substances [5,42]. Such ingredients may be used in food, cosmetic, and pharmaceutical industries in order to prevent oxidative damage and subsequent product deterioration [43]. To assess the impact of the addition of the seaweed *O. pinnatifida* extract to the antioxidant capacity of the cheese spreads, the quenching of the ABTS radical, DPPH free radical, and hydroxyl radical scavenging was assayed for the different cheese spreads (CSExt, CS, and CSFos) in day 0 and after 21 days of refrigerated storage. As can be seen in Table 2, in general, higher levels of antioxidant activity tested by ABTS radical, DPPH free radical, and hydroxyl radical scavenging assays were found for the cheese spread incorporating *O. pinnatifida* extract (CSExt) when compared with the other cheese spreads (CS and CSFos). Moreover, the CSExt showed similar percentages of ABTS activity throughout storage, i.e., in timepoints 0 and 21 days (*p* ˃ 0.05). In contrast, DPPH free radical and hydroxyl radical scavenging activities of the CSExt suffered a significant decrease throughout 21 days of refrigerated storage (*p* < 0.05). According to Rodrigues et al., *O. pinnatifida* extract using Viscozyme L showed antioxidant levels between 10% and 15%, around 4% and around 42% in ABTS radical, DPPH free radical, and hydroxyl radical scavenging assays, respectively [5]. In this alignment, the CSExt exhibited similar percentages of ABTS radical (13–15%) and DPPH free radical (3–5%) scavenging activities to those reported by Rodrigues et al. [5]. In contrast, the CSExt displayed higher percentages of hydroxyl radical scavenging (60–76%) than those described for the plain *O. pinnatifida* extract [5]. Together, these findings suggest that antioxidant activity is partially attributable to *O. pinnatifida* extract and that higher percentages of hydroxyl radical scavenging activity could be due to a synergistic interaction between components of the seaweed extract and the dairy matrix.

## 4. Conclusions

The present study is the first envisioning the development and characterization of a novel food containing *O. pinnatifida* extract as an ingredient. Indeed, when *O. pinnatifida* extract at 3% (*w*/*w*) was incorporated in a dairy matrix containing 75% (*w*/*w*) of whey cheese and 22% (*w*/*w*) of Greek-type yoghurt, it led to the formulation of an innovative food with high microbiological quality and interesting bioactivities. Microbiological analysis revealed the absence of microbial contamination of this cheese spread during 21 days of refrigerated storage. Furthermore, this product demonstrated prebiotic, antidiabetic, antihypertensive, and antioxidant properties immediately after manufacture and up to 21 days of storage, but in different magnitudes. Due to the commercialization potential of this novel food product, additional studies to confirm its nutritional composition and to monitor specific nutrient profiles or physicochemical parameters as well as consumer acceptance testing focused on specific sensory attributes should be performed.

## Figures and Tables

**Table 1:** Angiotensin I-converting enzyme (ACE) inhibition expressed in terms of the percentage (%) for *Osmundea pinnatifida* extract and the cheese spread incorporating *O. pinnatifida* extract (CSExt) at day 0 and after 21 days of storage.

Samples	ACE Inhibition Percentage (%) ^1^
*O. pinnatifida* extract	68.04 ± 3.70
CSExt at day 0 of storage	57.40 ± 1.52
CSExt after 21 days of storage	50.50 ± 0.28

^1^ Values are mean ± standard deviation (n = 3).

**Table 2:** Antioxidant capacities of the cheese spread incorporating *Osmundea pinnatifida* extract (CSExt), the cheese spread with fructooligosaccharides (CSFos), and the plain cheese spread (CS).

Cheese Spread	Storage (Days)	ABTS Radical Scavenging Activity (%) ^1^	DPPH Free Radical Scavenging Activity (%) ^1^	Hydroxyl Radical Scavenging Activity (%) ^1^
CSExt	0	14.34 ± 0.94 ^a^	4.47 ± 0.53 ^a^	74.71 ± 0.75 ^a^
	21	14.35 ± 0.46 ^a^	3.68 ± 0.58 ^a,^*	61.47 ± 1.90 ^a,^*
CSFos	0	9.91 ± 0.16 ^b^	3.20 ± 0.33 ^b^	70.59 ± 1.57 ^c^
	21	10.81 ± 0.17 ^b^	4.85 ± 0.51 ^b,^*	44.56 ± 0.01 ^c,^*
CS	0	10.15 ± 0.64 ^b^	2.78 ± 0.41 ^b^	68.07 ± 0.60 ^b^
	21	10.99 ± 0.36 ^b^	4.53 ± 0.24 ^b^	42.09 ± 0.41 ^b,^*

^1^ Results are expressed as mean ± standard deviation. Different letters indicate significant differences (*p* < 0.05) between cheese spreads at each storage time. The symbol * means statistically significant differences (*p* < 0.05) in comparison to the data obtained at day 0 of storage.

## Data Availability

The data are available from the corresponding author.
